# Conformation-independent structural comparison of macromolecules with *ProSMART*

**DOI:** 10.1107/S1399004714016241

**Published:** 2014-08-29

**Authors:** Robert A. Nicholls, Marcus Fischer, Stuart McNicholas, Garib N. Murshudov

**Affiliations:** a Structural Studies Division, MRC Laboratory of Molecular Biology, Francis Crick Avenue, Cambridge Biomedical Campus, Cambridge CB2 0QH, England; b Department of Pharmaceutical Chemistry, University of California San Francisco, San Francisco, CA 94158, USA; c Structural Biology Laboratory, Department of Chemistry, University of York, Heslington, York YO10 5DD, England

**Keywords:** *ProSMART*, Procrustes, structural comparison, alignment, external restraints, refinement

## Abstract

The identification and exploration of (dis)similarities between macromolecular structures can help to gain biological insight, for instance when visualizing or quantifying the response of a protein to ligand binding. Obtaining a residue alignment between compared structures is often a prerequisite for such comparative analysis. If the conformational change of the protein is dramatic, conventional alignment methods may struggle to provide an intuitive solution for straightforward analysis. To make such analyses more accessible, the *Procrustes Structural Matching Alignment and Restraints Tool* (*ProSMART*) has been developed, which achieves a conformation-independent structural alignment, as well as providing such additional functionalities as the generation of restraints for use in the refinement of macromolecular models. Sensible comparison of protein (or DNA/RNA) structures in the presence of conformational changes is achieved by enforcing neither chain nor domain rigidity. The visualization of results is facilitated by popular molecular-graphics software such as *CCP*4*mg* and *PyMOL*, providing intuitive feedback regarding structural conservation and subtle dissimilarities between close homologues that can otherwise be hard to identify. Automatically generated colour schemes corresponding to various residue-based scores are provided, which allow the assessment of the conservation of backbone and side-chain conformations relative to the local coordinate frame. Structural comparison tools such as *ProSMART* can help to break the complexity that accompanies the constantly growing pool of structural data into a more readily accessible form, potentially offering biological insight or influencing subsequent experiments.

## Abstract 2

The *Procrustes Structural Matching Alignment and Restraints Tool* (*ProSMART*) has been developed to allow local comparative structural analyses independent of the global conformations and sequence homology of the compared macromolecules. This allows quick and intuitive visualization of the conservation of backbone and side-chain conformations, providing complementary information to existing methods.

## 1. Introduction   

*Procrustes* was originally a code name for the program of Catell & Hurley (1962 ▶), named after the mythological Greek villain whose victims were stretched and cut in order to fit the shape of his bed:

Procrustes owned two beds, one small, one large; he made short victims lie in the large bed, and the tall victims in the short one

(Taleb, 2010 ▶). The ‘*Pro*’ in *ProSMART* (*Procrustes Structural Matching Alignment and Restraints Tool*) is owing to its use of Procrustes analysis (Gower & Dijksterhuis, 2004 ▶; Gower, 2010 ▶) for comparing local regions of structure between two protein chains. The analogy is fitting in this context owing to the manipulation of coordinates from one structure in order to optimally fit those in another, efficiently achieving a measure of local backbone r.m.s.d at a chosen level of structural resolution: the Procrustes score is effectively the minimal distance that one set of coordinates would have to move if forced to coincide with another. To clarify, by ‘local’ we mean a restricted contiguous structural environment close in space to a particular feature (*e.g.* a given residue/atom). Whilst there are many alignment tools that optimize a global superposition, there is a need for the development of methods that align macromolecular structures in a way that is independent of the global conformations of the compared chains. Such a facility could be exploited, allowing various complementary types of comparative structural analysis to be performed focusing on the conservation of local structure. Such analysis could reveal useful information that would otherwise be masked if using traditional comparison methods.

Comparative structural analyses are often performed in order to identify particular residues/regions that may be important for global/local fold stability or biological function, allowing the investigation of potential functional relationships and evolutionary links. Various approaches have been developed for the alignment and comparison of protein structures. These may be roughly classified as global methods, which require global spatial rigidity; flexible methods, which require piecewise spatial rigidity; and conformation-independent methods, which require only local structural conservation.

Traditional alignment methods have utilized various structural features, such as interatomic distances (Holm & Sander, 1993 ▶; Gerstein & Levitt, 1996 ▶; Aung & Tan, 2006 ▶), vectors (Taylor & Orengo, 1989 ▶; Ortiz *et al.*, 2002 ▶; Zhu & Weng, 2005 ▶), structural fragments (Alexandrov *et al.*, 1992 ▶; Shindyalov & Bourne, 1998 ▶; Konagurthu *et al.*, 2006 ▶; Pandit & Skolnick, 2008 ▶; Krissinel, 2012 ▶) and secondary-structure elements (SSEs; Gibrat *et al.*, 1996 ▶; Singh & Brutlag, 1997 ▶; Kleywegt & Jones, 1997 ▶; Szustakowski & Weng, 2000 ▶; Dror *et al.*, 2003 ▶; Krissinel & Henrick, 2004 ▶), with some methods also utilizing nonstructural information (Jung & Lee, 2000 ▶; Kawabata & Nishikawa, 2000 ▶; O’Hearn *et al.*, 2003 ▶). For a more detailed overview, see Nicholls (2011 ▶). The choice of feature used for analysis inherently affects the structural resolution of the comparison/alignment (note that this does not refer to crystallographic resolution, but rather to the level of structural detail). For example, the consideration of SSEs causes a reduction of information, resulting in the comparative analysis being performed at a much lower level of structural resolution (detail) than would arise from the direct utilization of atomic coordinates. Both low-resolution and high-resolution methods have their merits, being suited for identifying different types of similarity; there are benefits associated with using varying levels of structural resolution. High-resolution structural conservation (*e.g.* conserved side-chain positions) would only be expected for very similar structures, and thus may be used to distinguish between degrees of similarity within a class of close homologues. In contrast, low-resolution features (*e.g.* SSEs) would be very insensitive to such subtle dissimilarities owing to the inherent loss of detail, and would be more suited to identifying whether similar overall folds are adopted by nonhomologous or distantly related structures. It should also be acknowledged that lower resolution methods generally have the potential to be faster owing to using fewer landmarks to represent a structure.

It is worth noting that the term ‘alignment’ is often spuriously used synonymously with ‘superposition’, undoubtedly owing to the traditional prevalence of global alignment methods, which commonly achieve an alignment by optimizing a physical superposition. To clarify, here we refer to a structural alignment as identifying a correspondence between residues in two or more amino-acid sequences, derived using structural information, without any implication as to whether or not the aligned structures superpose well. An alignment is a discrete one-dimensional object that can be represented as a paired list of residue codes, without any reference to the three-dimensional structures: note that residues can be aligned without any structural information (*i.e.* sequence-based alignment). Indeed, the output of sequence-based alignment and structure-based alignment is qualitatively identical; the main difference between the two is the nature of the prior information. In contrast, a superposition is the three-dimensional overlay of the compared structures, which generally uses a given one-dimensional alignment as prior knowledge that specifies how the superposition should be optimized, noting that a one-dimensional alignment is not necessarily required to superpose structures (Vagin & Isupov, 2001 ▶).

For global methods, the structural alignment problem is generally considered analogous to that of fold recognition or rigid substructure identification, which exacerbates the commonly perceived ambiguity between the terms ‘alignment’ and ‘superposition’. Such methods often aim to identify the maximal list of residue/atom pairs that, when superposed, result in a measure of dissimilarity below some threshold, commonly the root-mean-square deviation (r.m.s.d.). This results in a reduction of the proportion of the chains being compared, resulting in scores corresponding to a substructure of size determined by some criterion. This backward-fitting results in the global r.m.s.d. score being largely arbitrary, and thus the number (or proportion) of aligned residues is often taken into account in order to achieve a more meaningful score (Subbiah *et al.*, 1993 ▶). Whilst such an approach can be useful in determining whether a conserved substructure is rigid and sizeable, it arguably has limited use in deriving measures for quantifying global dissimilarity of the compared chains in a more general sense. This is especially true when comparing similar structures that exhibit subtle or substantial differences in global conformation, which may be owing to effects that are biologically relevant such as binding, or owing to environmental factors such as crystal packing. It should also be acknowledged that the r.m.s.d. inherently depends on intrinsic properties of the structures being compared, for example size and globularity (Maiorov & Crippen, 1994 ▶).

In contrast, conformation-independent methods do not optimize global agreement and thus do not identify nor require the presence of rigid substructures (Morikawa, 2006 ▶). The intermediate flexible approaches search for piecewise rigidity, effectively taking a global approach but allowing the identification, alignment and superposition of multiple rigid regions, which is particularly useful in the presence of clear separable domain motion (Hayward & Berendsen, 1998 ▶; Shatsky *et al.*, 2002 ▶; Ye & Godzik, 2003 ▶; Schneider, 2004 ▶; Menke *et al.*, 2008 ▶; Mosca *et al.*, 2008 ▶; Shen *et al.*, 2010 ▶). Whilst most approaches focus on structural alignment, it should be noted that some flexible single-model rigid-substructure identification methods have been developed to address specific problems within the field of macromolecular crystallography (MX; Painter & Merritt, 2006 ▶; McCoy *et al.*, 2013 ▶). Even in the absence of multiple distinct domains, flexible approaches can provide different information to global methods whenever there is spatially correlated conformational flexibility. In this context, conformation-independent methods are even more powerful, being able to account for complex heterogeneous spatial movements and to detect structural conservation in regions that are locally conserved.

There are often distinct, measurable structural differences between highly homologous crystallographically determined macromolecular models. Such differences may occur at both global and local levels, which may be owing to biologically relevant factors or owing to the influences of crystal content and/or packing. Equally, it is often of relevance to analyse the structural variability of model ensembles achieved using other experimental or theoretical methods, such as electron microscopy (EM), nuclear magnetic resonance (NMR) spectroscopy and molecular-dynamics (MD) simulations. At the global level, structural differences include domain motion (for example owing to molecular binding), domain distortion (for example owing to crystal packing) and more dramatic conformational changes (for example domain swaps or alternative folds). At the local level, differences include changes in backbone and side-chain conformations, which may be subtle or dramatic and may or may not be of particular biological interest. Generally, identifying both regions that are and those that are not locally conserved can provide useful information during a comparative analysis. Such information cannot easily be inferred visually using a simple superposition and thus is often masked when using traditional representations. As such, the development of techniques dedicated to this task has been required: this demand motivated the development of *ProSMART*.

In this article, we first introduce structural fragments: the features chosen to represent local structure in *ProSMART*. We then describe the implemented alignment method, which uses a dynamic programming algorithm to achieve the solution. Following this, the conformation-independent comparative analysis features are described and the visualization of results is exemplified.

## 2. Conformation-independent structural alignment   

### 2.1. Structural fragments   

*ProSMART* uses structural fragments in order to represent the local structural environments of residues. The word ‘fragment’ has been used in various contexts within the field of molecular biology, such as in fragment-based ligand discovery. Interestingly, note that such chemical fragments are intended to break down chemical space, whilst structural fragments break down configuration space. Both represent the larger entity at a manageable degree of complexity.

Here, we consider a structural fragment to consist of the main-chain atoms N, C^α^, C and O from *n* consecutive residues (although other selections are allowed, for example only C^α^ atoms). *ProSMART* can deal with qualitatively different macromolecular structures (for example DNA/RNA) by using a different atom selection^1^. The fragment length *n* may be varied, allowing comparative analyses to be performed at different levels of structural resolution as desired (default *n* = 9). For purposes of subsequent residue-based backbone dissimilarity scoring, we require *n* to be odd, so that the central residue of a fragment always exists. Fragments are always complete and comparable, and only exist where there is a run of *n* valid residues. Structural fragments are represented as coordinate matrices. Consequently, these ordered constructs always comprise an equal number of directly comparable point landmarks (Dryden & Mardia, 1998 ▶), irrespective of the amino-acid sequences of the compared proteins (for example, if all protein backbone atoms are used a fragment always comprises 4*n* coordinates).

Importantly, the value of *n* is kept constant for all fragments during any single comparison: this allows the analysis to be performed at a single level of structural resolution, enabling objective comparison and interpretation of results. Repeating the analysis with different values of *n* allows a multi-resolution approach. Fragment indexing may be visualized using an *n*-residue sliding window along the protein chain constructed on a per-residue basis (fragments may overlap). For example, fragment 1 may comprise residues 1 to *n*, fragment 2 residues 2 to *n* + 1, and so on.

Further to being used in various structural alignment methods, structural fragments have also found use in hybrid structural–sequence methods, where the protein chain is represented as a one-dimensional sequence with alphabet determined by local fold classification, allowing fast alignment but utilizing a reduced amount of structural information (Friedberg *et al.*, 2007 ▶). However, in order to make objective comparison tangible and to prevent an excessive reduction of the available structural information, *ProSMART* avoids the separation of structural features into such discrete categories. It is for similar reasons that we do not choose to use SSEs for this application: such a representation would not allow the sensible comparison of any arbitrary chain pair, and such features may not be sufficiently well defined to be detectable in all cases.

### 2.2. Fragment-pair scoring   

In *ProSMART*, all pairs of structural fragments between two protein chains are compared by quantifying differences between the backbone atomic coordinates of the fragments. Specifically, we use a form of Procrustes analysis (Gower & Dijksterhuis, 2004 ▶; Gower, 2010 ▶) to describe differences in the pairwise distributions of fragment coordinates. The method is implemented so as to ensure invariance with respect to rigid-body transformations, *i.e.* translation and rotation of the original coordinate frames of the fragments. However, unlike the traditional implementations of Procrustes analysis, our implementation is purposely not scale-invariant as it is important to preserve atomic distances (our mythological Greek villain does not stretch the victimized fragments). Consequently, this is conceptually, but not practically, equivalent to coordinate-based superposition.

Despite being traditionally used in Procrustes analysis in the field of statistics, the procedure of optimally superposing two sets of coordinates is often referred to as the Kabsch or McLachlan algorithm in the field of biology (Kabsch, 1978 ▶; McLachlan, 1982 ▶). Whilst this method of superposition has been used in many alignment implementations, it should be noted that other approaches do exist, such as using the translation-independent u.r.m.s.d. (unit-vector root-mean-square deviation) (Chew *et al.*, 1999 ▶), minimizing the area between C^α^ traces (Falicov & Cohen, 1996 ▶) and superposing maps rather than coordinates (Vagin & Isupov, 2001 ▶).

Since Procrustes analysis provides a measure of dissimilarity that is invariant to rigid-body transformations, we ensure that the results are invariant to the global conformations of the macromolecules and to their original coordinate frames. In this context, the Procrustes score of a fragment pair is equivalent to the pairwise r.m.s.d. (root-mean-square deviation) of the corresponding atomic coordinates after superposition. The score thus represents the notion of the local r.m.s.d. about the central residues of the fragments. However, the use of Procrustes analysis allows the score to be realised without incurring as much computational expense as would arise from physically superposing the atomic coordinates and calculating the r.m.s.d. in the traditional fashion; this approach combines the benefits of high structural resolution with fast computation. We shall refer to the local r.m.s.d. score here as meaning the Procrustes distance.

In preparation for the structural alignment process, we calculate a dissimilarity matrix of the local r.m.s.d. scores of all fragment pairs between the two chains. Using the raw local r.m.s.d. means that the score is based on a natural measure of dissimilarity, being derived from minimization of the r.m.s.d. between fragment coordinate matrices **F**_1_ and **F**_2_ with respect to translation (**t**) and rotation (**R**) of **F**_2_,
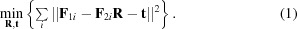
Without loss of generality, the translation component can be ignored by translation of the coordinate matrices to the origin. The local r.m.s.d. *d* between length 4*n* translation-normalized fragment coordinate matrices 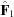 and 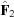 may then be efficiently calculated, 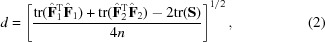
where **S** is the diagonal matrix of singular values obtained from the singular value decomposition of 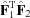 = **USV** ^T^, where **U** and **V** are 3 × 3 orthogonal matrices and tr denotes the trace of a matrix. As an aside, note that this formulation may be generalized to allow complex behaviour, such as coordinate weighting, repulsion and non-unique atomic correspondences (Nicholls, 2011 ▶).

This formulation is rotation-invariant, allowing quick calculation without needing to physically superpose (rotate) coordinate matrices in order to achieve the local r.m.s.d. When required, the corresponding rotation **R** ∈ *SO*(3) can be calculated (Challis, 1995 ▶),
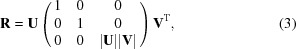
which is an orthogonal matrix with determinant unity, as required. Inclusion of the diagonal matrix ensures that the resulting matrix 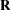 is indeed a rotation and not a rotoreflection. Note that if a rotoreflection is more favourable than a rotation (*i.e.* |**U**||**V**| = −1) then the fragments are most likely to be extremely dissimilar. This observation has been previously acknowledged and exploited for very long fragments (Maiorov & Crippen, 1994 ▶).

*ProSMART* uses data structures from the *TNT* package (Pozo, 1997 ▶) and a C++ translation of the *JAMA* package (Hicklin *et al.*, 2000 ▶) for computing the singular value decomposition of a matrix.

### 2.3. Fragment alignment   

The objective is to find the optimal fragment correspondence between the compared chains, according to minimization of the net local r.m.s.d. of aligned fragment pairs. Our approach imposes the constraint that the alignment must maintain sequence ordering (owing to our decision to use dynamic programming for alignment), which is deemed to be a suitable condition given our objective. Subject to this constraint, we aim to identify an alignment with the following desirable properties.

(i) Fragment alignment must be unique (one-to-one).
(ii) Alignment gaps are allowed, where appropriate.
(iii) The sum of aligned fragment dissimilarity scores is minimized.
(iv) Alignment length must be maximized.

One key feature of our approach is that the alignment length is maximized, regardless of the local feature-based scores of aligned fragments/residues. This ensures that the procedure is invariant to the choice of structural resolution of the analysis^2^.

Importantly, the alignment maximization ensures that the analysis pertains to the structural comparison of the whole chains, rather than just to specific portions of the chain (for example single domains). Indeed, the same alignment procedure is followed whether comparing structures that are similar or completely dissimilar. In line with our original intentions, an alignment will always be achieved between any chain pair, regardless of perceived structural similarity: dissimilarities will always be quantified regardless of the level of similarity between the compared structures. Nevertheless, the final residue alignment may optionally be filtered using a score threshold if it is desired for only regions considered sufficiently structurally similar to be aligned.

Fragment alignment comprises four main stages.

(i) Gap penalty assignment: to specify any bias (if any) to be included in the dynamic programming stage.
(ii) Dynamic programming: to achieve the optimal path subject to the maintenance of sequence order.
(iii) Path filtering: to identify the initial one-to-one consensus alignment between fragments.
(iv) Optimization: alignment is maximized and optionally refined, allowing potential for further improvement.

Being primarily interested in the conservation of local backbone structure, the method is completely independent of spatial relationships. However, following alignment, any similarities and/or spatial relationships can be subsequently identified and analysed.

#### 2.3.1. Dynamic programming   

In searching for the optimal alignment, it is impractical to consider all possible alignments, owing to combinatorial explosion. Rather, a dynamic programming algorithm is employed in order to find a reasonable but fast solution. Dynamic programming algorithms (Bertsekas, 2005 ▶) have commonly been used in biology for both sequence and structure-based alignment, as well as in other fields (Myers & Rabiner, 1981 ▶; Mongeau & Sankoff, 1990 ▶). The algorithm implemented in *ProSMART* is a modification of the Needleman–Wunsch dynamic programming algorithm (Needleman & Wunsch, 1970 ▶), similar to that used by other software tools such as *BLAST* (Altschul *et al.*, 1990 ▶). Integral to the method is the choice of input matrix that defines the similarity/dissimilarity of features. Such feature-based score matrices often inherit values from a smaller matrix of predetermined pairwise scores corresponding to particular states, often referred to as substitution or transition matrices (Dayhoff & Schwartz, 1978 ▶; Henikoff & Henikoff, 1992 ▶; Lo *et al.*, 2007 ▶). However, *ProSMART* adopts a continuous feature-based scoring scheme so that each feature pair is assigned a precise score^3^.

The objective of the dynamic programming algorithm implemented in *ProSMART* is to find the optimal path through the fragment dissimilarity matrix **D** beginning at position (1, 1) and ending at position (*N*_1_, *N*_2_), where *N*_1_ and *N*_2_ are the numbers of fragments in the compared protein chains. The algorithm optimizes the path through the dissimilarity matrix, resulting in the unique optimal one-to-many fragment correspondence between the two protein chains, such that the total sum of Procrustes scores is minimized.

We begin by constructing a cost matrix **C** to quantify, in terms of the cumulative local r.m.s.d. score, the costs associated with all paths through the matrix **D**. The elements of the cost matrix may be calculated recursively,
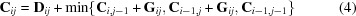
with the boundary conditions **C**_1*j*_ = **D**_1*j*_ and **C**_*i*1_ = **C**_*i*1_. Here, the matrix **G** specifies any gap penalties applied to the nonalignment of fragment pair (*i* − 1, *j* − 1) given the alignment of pair (*i*, *j*). The optimal path *P*, which has the minimum associated cost, may be calculated recursively backwards,
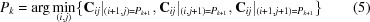
beginning with the boundary condition *P*_|*P*|_ = (*N*_1_, *N*_2_).

The employed approach allows a gap penalty to be assigned to nonconsecutive alignments. However, *ProSMART* does not use a general gap penalty in the conventional sense. By avoiding the use of a gap penalty, we avoid unnecessarily introducing arbitrary parameters, ensuring that insertions and deletions are dealt with unambiguously. Nevertheless, more complex gap penalties may be used where required, for example to ensure the consecutive alignment of repetitive helical fragments in the presence of noise.

#### 2.3.2. Path filtering   

The path *P* specifies the optimal one-to-many correspondence between fragments. However, we require a one-to-one correspondence so that a given fragment cannot be aligned to multiple fragments in the other protein chain. Despite the fact that the dynamic programming solution may not necessarily realise the optimal alignment subject to our criteria, it generally gives a very good approximation to the solution and suffices well to provide an initial alignment.

At this stage, the path *P* is filtered so that any fragment from one chain is aligned to at most one fragment from the other chain, keeping the fragments that score most favourably. The initial one-to-one fragment alignment *A* is constructed thus:
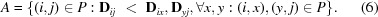
Since this one-to-one fragment alignment does not necessarily imply a one-to-one residue correspondence, further alignment optimization is required.

#### 2.3.3. Final alignment optimization   

A more favourable alignment is achieved by minimizing the net local r.m.s.d.,
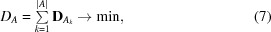
subject to an implied unique one-to-one residue correspondence and the alignment length being maximized (*i.e.* any unaligned fragment pairs cannot be aligned without violating other conditions).

Various (optional) stages are implemented in order to iteratively approach the solution. Alignment is first refined without and subsequently with enforcing an implied one-to-one residue correspondence. The procedure involves trialling deterministic permutations to the fragment correspondence, searching for alignments with lower *D_A_* whilst maintaining an equal length |*A*|. After each round of iterative optimization, the alignment is lengthened where possible in order to maximize |*A*|. Note that this procedure cannot worsen the alignment (in terms of our criteria). The added computational expense is justified owing to being orders of magnitude faster than the overall alignment procedure. For details of this procedure, see Nicholls (2011 ▶).

## 3. Conformation-independent dissimilarity scoring   

Often, it can be hard to identify or quantify subtle differences between models, especially when attempting to do so by simply superposing structures and inspecting them manually. This can be even more challenging when the compared models cannot be easily/unambiguously superposed, such as when the models undergo conformational change. However, this task can be made dramatically easier by investigating the conservation of local structure, which can provide great insight.

*ProSMART* reports various residue-based local dissimilarity scores which pertain to the conservation of backbone and side-chain conformation. Results regarding alignment, superposition, rigid substructure identification and residue-based scoring may be visualized using the popular molecular-graphics software *CCP*4*mg* (McNicholas *et al.*, 2011 ▶) and *PyMOL* (Schrödinger). Residues are coloured using an intuitive gradient (colours and gradient scales may be chosen) representing various levels of dissimilarity. This default output can provide useful information that can be hard to achieve manually and at the same time easily and automatically produce publication-quality graphical representations of structural analyses. In particular, the *ProSMART* interface within *CCP*4*mg* offers powerful functionalities, including the ability to alter colours and gradients in real time.

The examples in this section involve publicly available models of macromolecular structures deposited in the Protein Data Bank (Berman *et al.*, 2002 ▶). These models are referenced using their PDB IDs and chain identifier codes. All examples correspond to default *ProSMART* comparison using a fragment length of nine residues.

### 3.1. Residue-based local backbone conservation scores   

We exploit the fact that a particular residue may belong to multiple aligned fragments by identifying multiple ways of scoring. Referred to as the Procrustes, Flexible and Hinging scores^4^, these scores provide complementary information and can be used in concert to analyse the local structural environments of residues.

**Figure 1:**
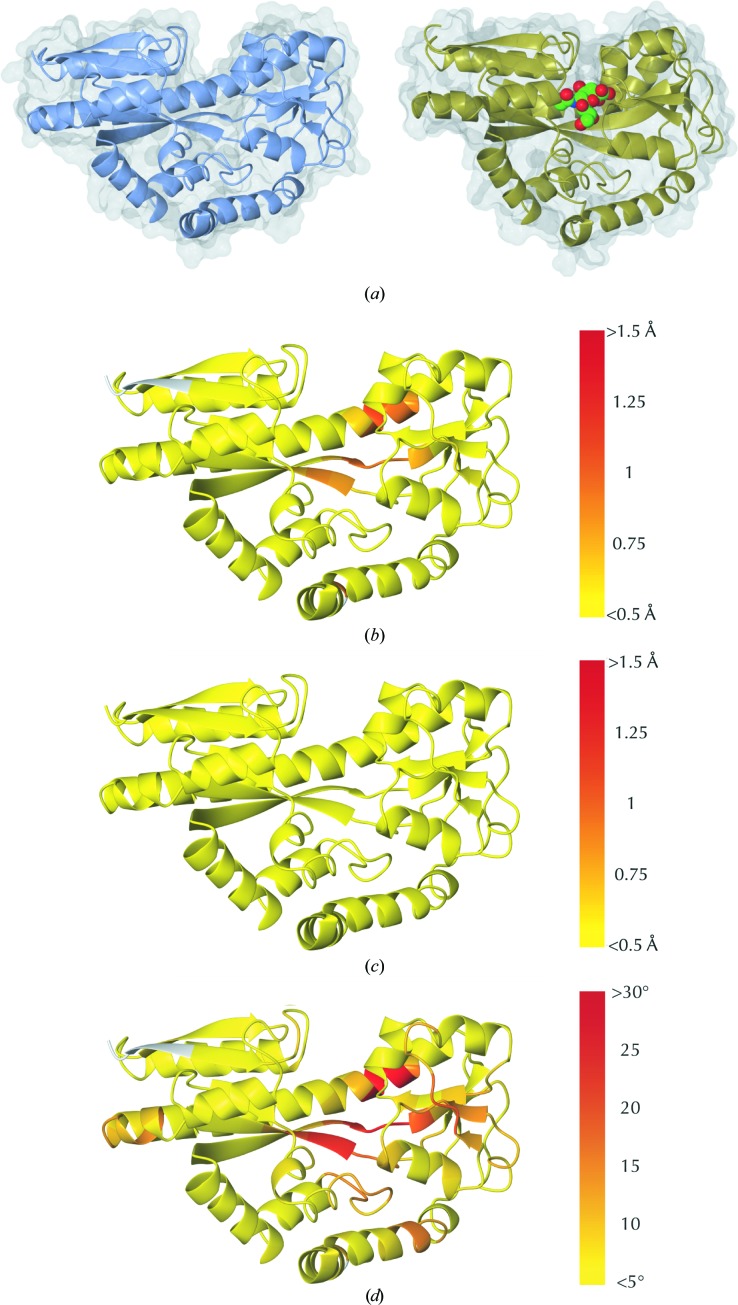
Structural comparison of the backbone in the presence of ligand-induced conformational changes. Illustrations of results from the default *ProSMART* comparison of open (PDB entry 2cex chain *A*) and closed (PDB entry 3b50 chain *A*) forms of the SiaP TRAP sialic acid-binding protein, coloured using a colour gradient according to main-chain dissimilarity scores (yellow implies similarity and red relative dissimilarity; white, not applicable). Since the two models do not superpose well, for clarity only 2cex chain *A* is shown in (*b*)–(*d*). The Procrustes score (*b*) allows easy identification of locally distorted regions (such as hinges). The Flexible score (*c*) helps to identify regions that are at all similar, despite any global conformational change (note that the whole structure is coloured yellow, indicating high local similarity despite different global conformations). The Hinging score (*d*) is useful for identifying subtle backbone deformations (including hinges) that can otherwise be very hard to identify. These complementary depictions allow quick visual identification of exactly which regions are structurally very similar and which exhibit differences. (*a*) Open (2cex chain *A*, left) and closed (3b50 chain *A*, right) forms of SiaP. (*b*) Coloured by the Procrustes score. (*c*) Coloured by the Flexible score. (*d*) Coloured by the Hinging score.

(i) The Procrustes score of a residue is inherited from the fragment centred on that residue. This represents a one-to-one map between aligned fragments and residues; only residues located at the centre of an aligned fragment have an assigned Procrustes score. This score, which is directly used in the alignment procedure of *ProSMART*, measures the raw structural dissimilarity of the immediate local backbone environments of the residues (see Fig. 1 ▶ *b*).
(ii) The Flexible score of a residue is inherited from the best-scoring aligned fragment pair that the residue belongs to (residues may belong to up to *n* aligned fragments). This can be conceptualized as a sliding window of fragments passing over the residue; the Flexible score of a residue is the Procrustes score corresponding to the best-matching aligned fragment pair. As such, the Flexible score is highly insensitive to global conformation. Residues with a low (good) Flexible score are expected to belong to conserved regions between the compared structures (see Figs. 1 ▶ *c*, 2 ▶, 3 ▶ *a* and 4 ▶). In contrast, residues with a low Procrustes score (Fig. 1 ▶ *b*) are expected to be well embedded within conserved regions.
(iii) The Hinging score represents the degree of rotational hinging of the backbone about the central residue, being highly sensitive to any backbone curvature or torsion. This score only exists for residues that are central to an aligned fragment, and is consequently particularly useful for identical or near-identical structures. Allowing identification of regions that exhibit any subtle backbone deformations, the Hinging score provides useful information that can aid in the identification of individual residues involved in conformational change (Fig. 1 ▶ *d*).

**Figure 2:**
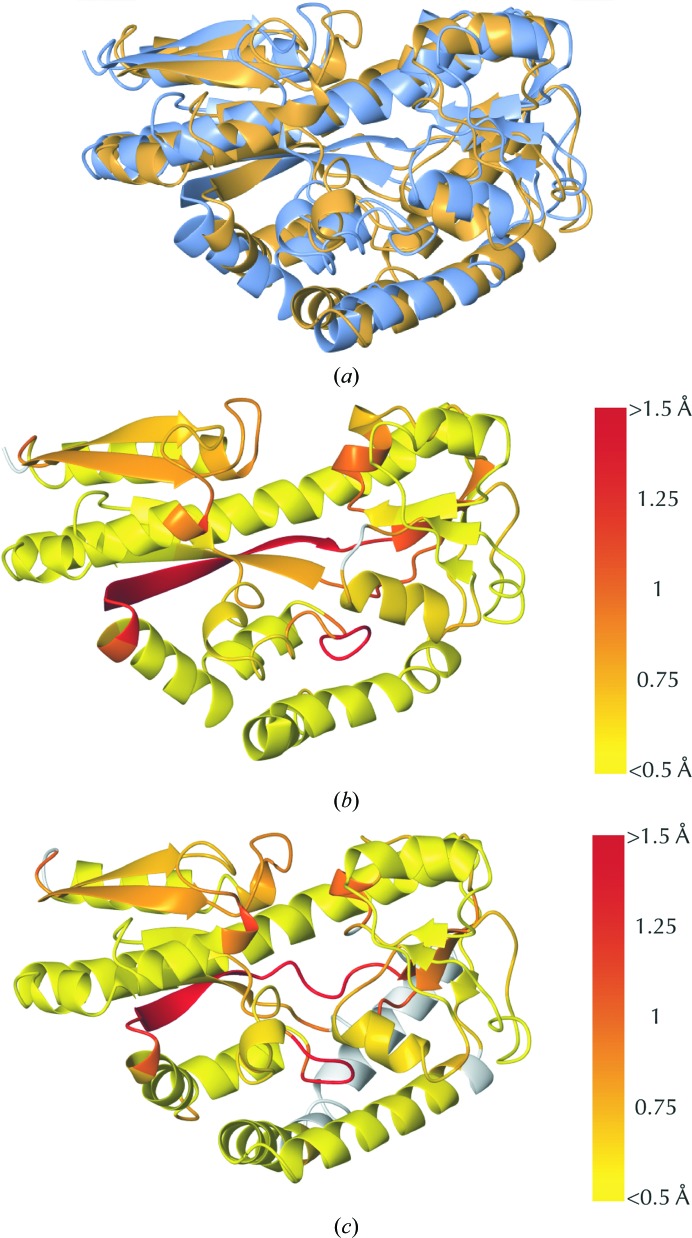
Comparison of structures sharing low sequence homology. *ProSMART* structural comparison of a sialic acid-binding protein (PDB entry 2cex chain *A*) and a sodium α-keto acid-binding protein (PDB entry 2hzk chain *A*), which share only 14% sequence identity despite exhibiting the same overall global fold. In (*b*) and (*c*) the models are coloured by the Flexible score using a colour gradient (yellow implies similarity and red relative dissimilarity; white, not applicable). This representation allows quick and easy visual identification of exactly which regions are structurally similar and which exhibit differences; note that this level of insight could not be achieved by simple superposition (*a*). (*a*) 2cex chain *A* and 2hzk chain *A* superposed. (*b*) 2cex chain *A* coloured by the Flexible score. (*c*) 2hzk chain *A* coloured by the Flexible score.

Of the three backbone scores, the Flexible score is often of most practical interest and thus is always recommended as a first port of call, especially when comparing structures that are not near-identical in sequence. For example, in Fig. 2 ▶ it is evident that local backbone structure is preserved in many regions (coloured yellow), including many loops, despite the low sequence homology between the compared structures. Simultaneously, it is easy to identify which regions are structurally dissimilar (coloured red).

**Figure 3:**
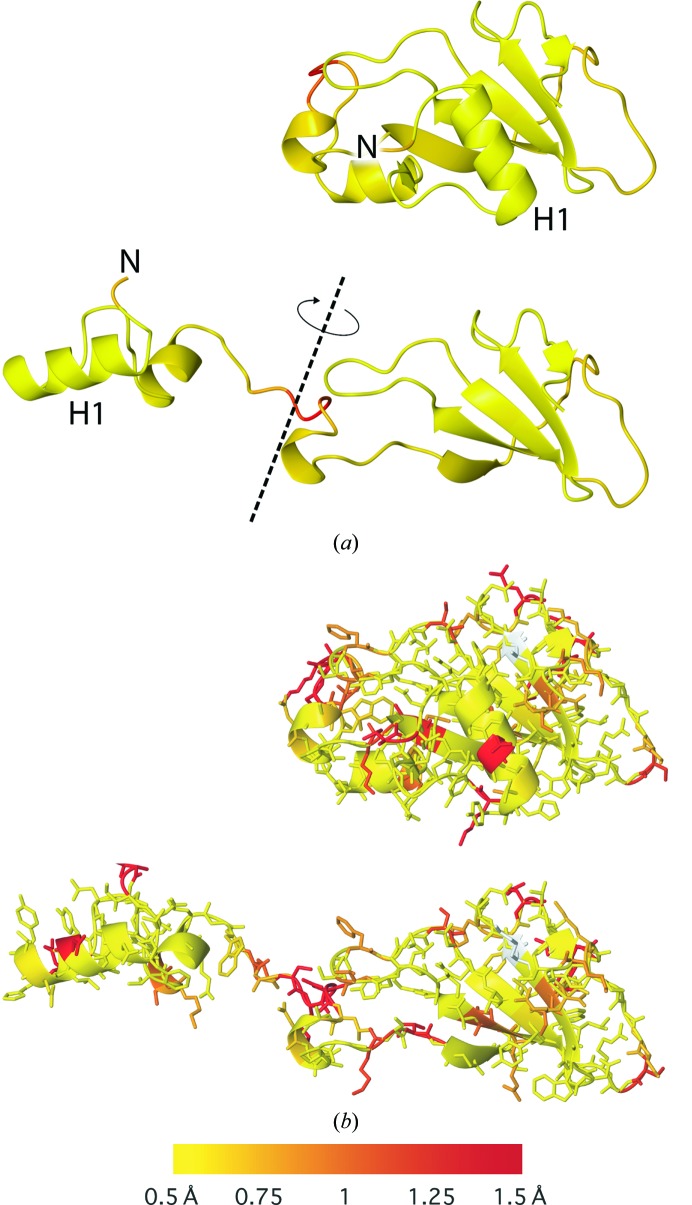
Conformation-independent structural comparison in the presence of domain swaps. Models of barnase with different biological assemblies are compared; the model 1yvs chain *A* corresponds to the trimeric domain-swapped form, unlike the sequence-identical model 2za4 chain *A*. To help illustrate the nature of the conformational change, in (*a*) the N-terminus is labelled N and the N-terminal helix is labelled H1. The models are coloured by (*a*) the *Flexible* score and (*b*) the side-chain r.m.s.d. score using a colour gradient (yellow implies similarity and red relative dissimilarity; white, not applicable). This demonstrates the ability to analyse structural conservation despite the presence of large conformational changes such as domain swaps, noting that this approach does not require spatial relationships to be conserved nor domains to be intact; only the conservation of local structure is of relevance. (*a*) Flexible score: 1yvs chain *A* (top) and 2za4 chain *A* (bottom). (*b*) Side-chain r.m.s.d. score: 1yvs chain *A* (top) and 2za4 chain *A* (bottom).

The Flexible score can also help to identify whether structural regions are internally near-identical, even if the compared models adopt dramatically different global conformations. For example, Fig. 3 ▶ shows an example of comparing a model of barnase with a domain-swapped form. By simply looking at the structures manually, it can be hard to determine the degree to which the domain swap induces conformational change of the backbone. Colouring the models using the Flexible score (Fig. 3 ▶ *a*) reveals that the backbone is highly structurally preserved everywhere except for the few residues involved in the hinge (which are coloured red).

### 3.2. Comparison of side-chain conformations   

Further to scores describing the backbone structural dissimilarity, *ProSMART* provides various measures of the conformational conservation of side chains relative to their local coordinate frames, including the side-chain r.m.s.d. score and the maximal deviation over all atoms in the residue. These scores only make sense for sequence-identical residues with low Flexible scores. The side-chain r.m.s.d. is the average distance between corresponding side-chain atoms in the target residue pair after local superposition. When comparing non-identical structures with high structural similarity (for example mutants) the distance between average positions of side-chain atoms may be used, as it can be calculated for residues with different amino-acid types. Results should be interpreted contextually, remembering that side chains from different amino acids will have different score distributions.

This functionality may be used to compare close homologues whether in the same or different global conformational states. For example, in Fig. 3 ▶(*b*) consideration of the side-chain r.m.s.d. score allows the immediate visual location of side chains that adopt similar and different conformations in the two models. This can be useful in various situations, for example to investigate and visualize differences in side-chain conformation in sites of interest or the effects of external influences such as small-molecule and metal binding, biological assembly and crystal packing.

### 3.3. Application of comparative structural analysis in crystallographic model building and refinement and in other fields   

*ProSMART* comparative structural analysis can also be used in model refinement, allowing comparison of models at various stages in the refinement process, including the quick visual identification of subtle differences between NCS-related chains. Comparing a model using *ProSMART* before and after crystallographic refinement allows investigation into the extent of any local backbone and/or side-chain conformational changes that occur during refinement. This can provide information regarding stability during refinement, the effect of different refinement protocols and the degree of influence of any external restraints used (Nicholls *et al.*, 2012 ▶). Such information can be used to gain intuition regarding stability during refinement and the usefulness/suitability of different protocols (for example, the use of external restraints) and consequently be used to hone the refinement process. For example, it may be desirable to use external restraints for some regions/residues but not for others. It is often tedious to systematically manually inspect every residue to see where the model has changed. Using the presented *ProSMART* comparative analysis features, it is possible to quickly and easily identify which regions are likely to be in the most drastic need of attention.

**Figure 4:**
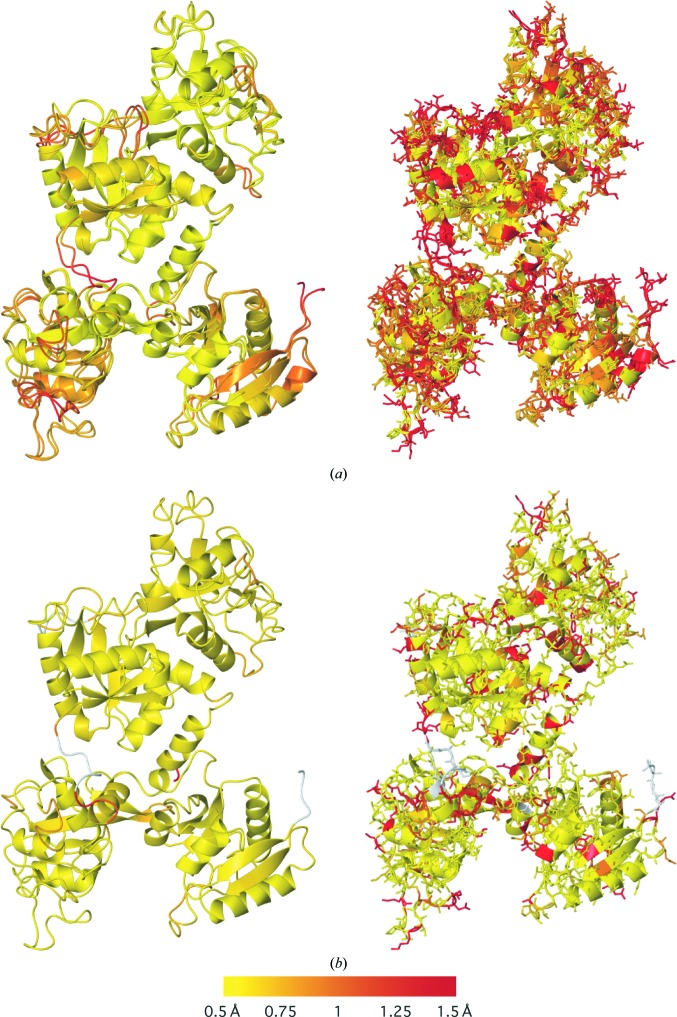
Application of comparative structural analysis in crystallographic model building and refinement. Comparative analysis of the backbone (left) and side chains (right) of (*a*) the 3.5 Å resolution model 1ryx of ovotransferrin before and after re-refinement with external restraints from the sequence-identical 2.15 Å resolution model 2d3i and (*b*) 1ryx after re-refinement using external restraints and the reference model 2d3i. For clarity, the reference model 2d3i is not shown. The models are coloured according to the Flexible backbone score (left) and the side-chain r.m.s.d. score (right) using a colour gradient (yellow implies similarity and red relative dissimilarity; white, not applicable). (*a*) Comparison of 1ryx before and after re-refinement (superposed). (*b*) Comparison of 1ryx after re-refinement and 2d3i (not shown).

Fig. 4 ▶ illustrates an example of comparing a model re-refined using external restraints against the original structure (Fig. 4 ▶ *a*) and the reference structure from which the external restraints were generated (Fig. 4 ▶ *b*). The model of 1ryx was re-refined using *REFMAC*5 (Murshudov *et al.*, 2011 ▶). Details of the re-refinement of 1ryx using 2d3i as a reference structure are given elsewhere (Nicholls *et al.*, 2013 ▶). From Fig. 4 ▶(*b*) (left) we can see that the backbone of the target structure has been pulled towards the conformation of the reference structure during refinement: the two models have locally similar backbones. However, there are a few regions that have not been pulled into the conformation of the reference structure. By visual comparison we can see that the backbone of the re-refined model is locally more similar to the reference structure (Fig. 4 ▶ *b*, left) than to the original target model (Fig. 4 ▶ *a*, left). Also, it is evident that there are a substantial number of side chains that adopt different conformations in the re-refined and reference models (Fig. 4 ▶ *b*, right). This demonstrates that, despite using external restraints on all side chains, the external restraints have not pulled side chains out of their conformation where the density is strong enough to suggest that they should stay where they are. The analysis also allows the assertion that more side chains adopt the conformation of the reference structure (Fig. 4 ▶ *b*, right) than the conformation of the original target structure (Fig. 4 ▶ *a*, right). Such analysis can be useful in investigating whether restraints are too tight or too loose, providing insight that might be used to hone the refinement protocol.

**Figure 5:**
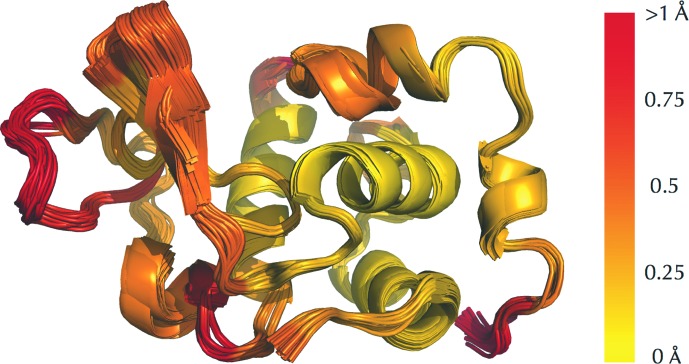
Processing ensembles from other methods, such as from NMR spectroscopy. Multi-model comparative analysis of a solution NMR structure of hen egg-white lysozyme (PDB entry 1e8l). All models are coloured using the same scheme, using a colour gradient (yellow implies similarity and red relative dissimilarity; white, not applicable). Residues in the ensemble are coloured according to the maximum (worst) Flexible score over all models in the ensemble, using the first model as the target.

In addition to being used for the comparative analysis of crystallographically derived macromolecular models, *ProSMART* can also be used to compare structures resolved using other experimental methods. For example, Fig. 5 ▶ demonstrates the internal comparison of the backbone of an NMR ensemble using the Flexible score. Considering the ensemble superposition alone, it is often difficult (if not impossible) to discern which regions are internally rigid and which are locally flexible. By colouring according to local backbone conservation, it is much easy to identify the rigid core (coloured yellow, right of the middle) in contrast with the relatively more mobile surface regions (coloured red). Note that the colour gradient used is different to that in other figures, demonstrating how different gradients can be useful in different contexts.

**Figure 6:**
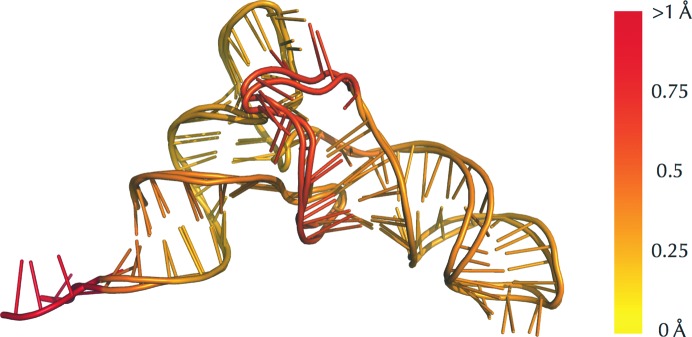
Structural comparison of nucleic acids. Local comparative analysis of the P-site and E-site fMet-tRNA models from a 70S ribosome (PDB entry 3d5a chains *Y* and *Z*). The models are superposed and coloured according to the Flexible backbone score using a colour gradient (yellow implies similarity and red relative dissimilarity). The C1′, C4′, O3′ and O5′ atoms were used for the comparison in rough analogy with the four backbone atoms in proteins (any selection of atoms common to all nucleotides might have been selected). This allows straightforward visual identification of regions with low structural conservation (centre and left), whereas other regions (right) might visually appear dissimilar when superposed but are actually more conserved locally. This highlights how *ProSMART* can provide complementary information that cannot be readily achieved simply by looking at superposed structures or considering r.m.s.d. values.

In addition to protein chains, the *ProSMART* method can also be used to compare other coordinate-based objects that correspond to ordinal sequences, such as nucleic acids (Fig. 6 ▶). Such analyses may be performed using models derived using various techniques (*e.g.* MX or EM; Brown *et al.*, 2014 ▶).

## 4. Discussion   

The conformation-independent structural comparison tool *ProSMART* (*Procrustes Structural Matching Alignment and Restraints Tool*) is designed to allow fast but detailed comparative analysis of macromolecular structures in the presence of conformational changes. *ProSMART* is suited to the analysis of the structural conservation of local backbone and side chains in a wide variety of scenarios: the method is sensitive enough to allow identification of subtle dissimilarities between structures sharing high sequence homology, whilst being versatile enough to scale to the identification of surprising local similarities between more distantly related structures.

*ProSMART* compares local structures using *n*-residue backbone fragments. These constructs allow comparisons to be performed at a chosen level of structural resolution or at multiple resolutions (note that this does not refer to crystallographic resolution, but rather to the level of structural detail). In contrast with most other features (*e.g.* SSEs) there is potential for structural resolution to be chosen in a relatively smooth fashion, since the fragment length may be selected as desired. In more detailed studies, a multi-resolution analysis may be performed by considering a variety of fragment lengths. This can provide useful and complementary insight regarding conformational differences between the compared models, allowing a rich breadth of information to obtained that may be used to more closely examine the nature of any observed (dis)similarities. For example, choosing a short fragment length (3–5 residues) results in performing analyses at a high level of structural resolution, which could be useful for the highly sensitive analysis of local backbone curvature in hinge regions. In contrast, choosing a long fragment length (>9 residues) would operate closer to the secondary-structure level, smoothing out any finer details and providing a more stable lower resolution view, whilst being more affected by larger conformational differences between the compared structures. Default analysis would typically be performed using intermediate fragment lengths (7–9 residues), offering a reasonable tradeoff between sensitivity, stability and conformation-independence.

Alignment and comparison by *ProSMART* is fast, typically taking a fraction of a second to align and compare an average-sized chain pair. This is owing to the use of Procrustes analysis for calculating local r.m.s.d., rather than superposing structures in the conventional fashion. Furthermore, *ProSMART* utilizes parallel environments, allowing multiple chain pairs to be simultaneously co-processed.

As a method, structural alignment by *ProSMART* can be thought of as complementary to traditional alignment methods. The approach is intended to provide a representation that is unique and useful. Consequently, owing to having markedly different objectives, *ProSMART* cannot be meaningfully compared with other available tools. Indeed, *Pro­SMART* does not attempt to optimize a superposition nor to address the fold-recognition problem, which are the objectives of most existing alignment methods. Rather, *ProSMART* aims to align the backbone in a way that optimizes the net agreement of local structures along the chain, allowing a sensible method of alignment that is completely independent of the global conformations of compared models at the chosen level of structural resolution. This method does not require the presence of domains nor other rigid structural units, and in fact is indifferent to whether any spatial relationships are conserved. By design, the method has the noteworthy limitation that chains exhibiting the same global fold but no conservation of local structure cannot be meaningfully aligned/compared in this way (other than to clarify that local structure is not conserved).

One key feature of the approach is that the alignment length is always maximized, regardless of the magnitude of scores corresponding to aligned residues. This ensures that the procedure is invariant to the choice of structural resolution (*i.e.* fragment length) and to the nature of the structures being compared (for example, protein *versus* DNA/RNA). Furthermore, this ensures that the analysis pertains to the structural comparison of the whole chains rather than just to specific portions of the chain (for example single domains) and allows the exact same alignment procedure to be used whether comparing structures that are similar or completely dissimilar. Importantly, an alignment will always be achieved between any chain pair, regardless of perceived structural similarity: dissimilarities will always be quantified regardless of the level of similarity between the compared structures. As an extreme example, note that an all-α structure may be aligned with an all-β structure: in this case a long alignment will be achieved and poor residue-based scores will be realised. Of course, if such behaviour is deemed undesirable then the alignment may be filtered using a score threshold, resulting in an alignment in which all aligned regions are sufficiently similar; *ProSMART* is highly customisable, providing the user with the ability to have a more bespoke experience if desired. If difficulties are experienced aligning proteins of dissimilar size, it may be because there is a lack of local backbone structural conservation between the compared models or alternatively there may be multiple regions in the larger model that are approximately equally locally similar to a given region in the smaller model. In such cases, it is recommended to try adjusting the chosen level of structural resolution (for example specifying that only C^α^ atoms are used for alignment or changing the fragment length) or alternatively to specify particular residue ranges to be aligned.

The comparative analysis features of *ProSMART* have potential to be useful for a wide variety of purposes, providing the ability to analyse structures at varying levels of detail. For example, near-identical models may be compared at a very high level of detail, investigating subtle differences between corresponding backbone regions or side chains. This could be used to investigate the influence of different environmental conditions (for example, different ligand-binding modes, different crystal contacts *etc.*) or to assess the extent of the changes a model undergoes during the crystallographic model-building and refinement process. Comparative structural analysis at more moderate levels of detail may be performed on highly homologous structures, often those which adopt slightly or substantially different global conformational states. Evaluation of such conformational changes may involve the identification of residues involved in conformational change, description of any hinging motions and assessment of internal surface-loop variability. At a lower level of detail, the backbone scores provided by *ProSMART* are able to distinguish between varying levels of local dissimilarity, irrespective of the overall similarity between the compared structures. In practice, this can be useful for the identification of local similarities between seemingly dissimilar structures and the visualization of local dissimilarities in corresponding regions of homologous structures. Other comparative analysis functionalities available in *ProSMART* that are not conformation-independent, such as methods of rigid substructure identification and superposition, are beyond the scope of this article and will be discussed elsewhere.

The provision of various residue-based local dissimilarity scores for the backbone and side chains, and the ability to intuitively view results in colour using the molecular-graphics software *CCP*4*mg* (McNicholas *et al.*, 2011 ▶) and *PyMOL* (Schrödinger), allows a unique and informative way of performing comparative structural analyses. The information provided can be useful as it is often masked when using traditional representations (*i.e.* it cannot be easily inferred visually using a simple superposition). At the same time, the output easily allows the production of quality graphical representations of structural analyses. In particular, the *ProSMART* interface within *CCP*4*mg* offers useful functionalities, including the ability to alter colours and gradients in real time.

*ProSMART* has diverse application in the analysis of the models of protein and DNA/RNA structures, accepting models available in PDB format resolved using a variety of techniques (*e.g.* MX, EM, NMR and MD). In addition to being used for comparative structural analysis, *ProSMART* also allows the generation of interatomic distance restraints for use in MX refinement. The adopted alignment approach is considered to be appropriate for this application since the generated restraints operate locally, being independent of global conformational differences between the target and reference structures. This functionality has been described previously (Nicholls *et al.*, 2012 ▶).

It should be acknowledged that the usefulness and limitations of structural comparison are dependent on the quality of the compared models. Whilst we often assume a reasonable degree of experimental reliability and accuracy, the potential for model errors should not be overlooked. Indeed, some deposited models have been found to be incorrect (Chang, 2007 ▶; Bujnicki *et al.*, 2002 ▶), and even those that are considered to be correct cannot be considered to be perfect, as suggested by the improvements observed from the re-refinement of deposited models (Joosten *et al.*, 2009 ▶).

The fact that crystallographically derived models have errors is often overlooked when performing structural analyses. It is important to remember that whilst atomic coordinate data are static, macromolecules are actually dynamic in nature. Note that models are averaged over the range of conformations present in the heterogeneous crystal, which comprises a practically infinite ensemble of structures. This is reflected by positional uncertainty (parameterized as *B* factors) and, in the case of more extreme flexibility, missing atoms (disorder). Furthermore, model reliability may vary: some models may exhibit substantial incorrect regions, depending on data quality, crystallographic resolution and the presence of modelling errors.

Consequently, there would be a temptation to account for model uncertainty when attempting to perform structural analyses using crystallographically derived models. For instance, one attempt to account for structural reliability might involve weighting coordinates according to a measure of positional uncertainty. However, such a method would be flawed owing to failing to account for the correlated motion of close atoms: such an approach would result in a measure of positional uncertainty relative to the coordinate frame of the crystal lattice and not necessarily a measure of local conformational flexibility (as would be required). Note that model errors in the form of atomic uncertainties are purposefully not taken into account as part of *ProSMART* comparative analysis owing to this ambiguity of interpretation. This is further justified by the use of restraints in crystallographic refinement, which ensure that local structures within the model adopt chemically sensible conformations.

With this in mind, it should be mentioned that model reliability should ideally be considered (for example by inspection of the electron density) when performing any sort of structural analysis, remembering that the result of a structural comparison is simply a narrative, requiring a succinct contextual interpretation in order to be meaningful. When performing any structural bioinformatics work, it is often beneficial to acknowledge that the static models under consideration are not flawless: experimentally derived models have errors and are in fact imperfect averaged snapshots of a dynamic structure. Whilst thermal parameters are often available (whether or not they are reliable), such description is a gross simplification of the actual system and does not capture information regarding true conformational variability.

Owing to the ever-increasing number of structures (and thus information) in the PDB available for exploitation, as time progresses there will be an increasing need for the provision of tools that allow easy navigation and extraction of relevant information. It seems reasonable that at some point the number of new structures/folds discovered will diminish and the amount of truly unique structural information available will begin to saturate (Chothia, 1992 ▶). At such a point, the main challenge encountered by structural biologists may shift from experimental structure determination to navigation of data and extraction of information, which would undoubtedly heighten the necessity for effective and varied methods of comparative structural analysis.

Owing to the vastness of protein conformational space, there is great potential for many types of comparative structural analyses to be performed. For example, the identification of subtle or non-obvious structural links between seemingly unrelated structures may provide useful information regarding the evolution, function or structural stability of proteins at the local and/or global level. At the same time, as the PDB grows and structural redundancy increases (*i.e.* many isomorphous or homologous models are deposited), there will be increased demand for the comparative analysis of very similar structures, for example in order to provide deeper insight regarding intra-class variability and the effect of external influences. Consequently, structural bioinformatics will undoubtedly become even more relevant in future.

*ProSMART* is available under the GNU LGPL v.3 open-source license (http://www.gnu.org/licenses/) as a standalone package (http://www2.mrc-lmb.cam.ac.uk/groups/murshudov/), as well as being distributed as part of the *CCP*4 suite (http://www.ccp4.ac.uk/; Winn *et al.*, 2011 ▶), and can currently be executed either as a command-line tool, through the *CCP*4*i* GUI (Potterton *et al.*, 2003 ▶) or *via CCP*4*mg*.

## Footnotes

1 For example, in the case of DNA/RNA any of the atoms common to all nucleotides may be selected for use as directly comparable landmarks, for example C1′, C4′, O3′ and O5′ could be selected by rough analogy with the four backbone atoms in proteins. Another choice might be the P atom, by analogy with the C^α^ atom.

2 Note that the comparison of longer fragments will systematically increase the local r.m.s.d. score, yet the raw alignment approach is agnostic to score magnitude.

3 Note that other methods have used different measures to populate the score matrix, for example interatomic distances (Subbiah *et al.*, 1993 ▶; Gerstein & Levitt, 1996 ▶).

4 Note for clarity: in the *ProSMART* output and documentation, the Procrustes, Flexible and Hinging scores have previously been termed the Central, Minimum and Rotate scores, respectively.
